# Supplementary material for: Apoplastic effector candidates of a foliar forest pathogen trigger cell death in host and non-host plants
Source: Sci Rep. 2021 Oct 7;11:19958. doi: 10.1038/s41598-021-99415-5 (PMC8497623; doi:10.1038/s41598-021-99415-5)
Supplement: Supplementary file 1 — Supplementary Figures. [file 41598_2021_99415_MOESM1_ESM.pdf]

Hunziker et al. Supplementary Figures S1-S4

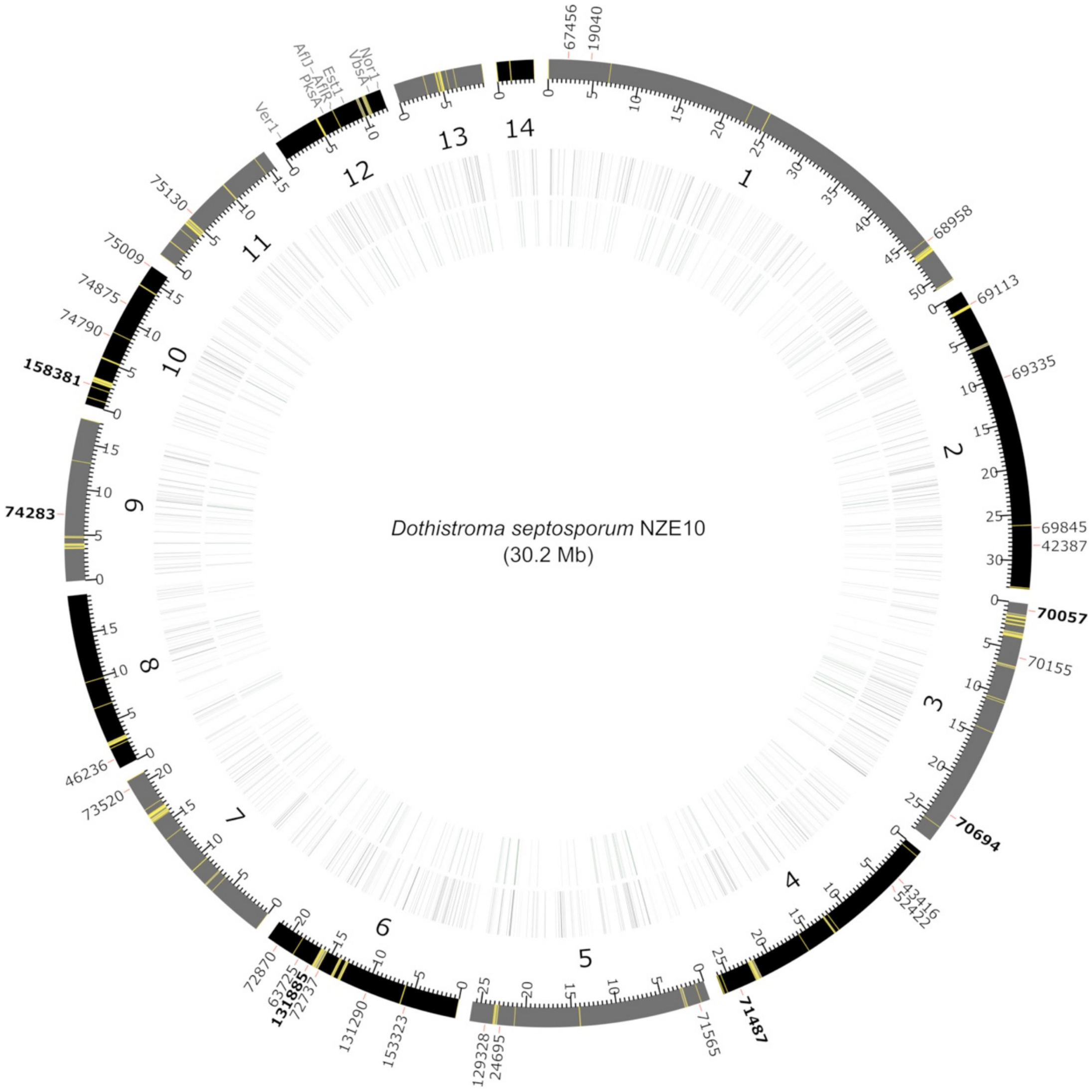

Figure S1: Locations of the 30 cloned *Dothistroma septosporum* candidate effector (DsCE) genes in the NZE10 genome. The 14 chromosome-level scaffolds of *D. septosporum* NZE10 [1] are represented by the outer bars. Each minor tick represents 5,000 bp from the start of the scaffold; yellow stripes indicate the location of curated repetitive elements >200 bp in length [2]. Outer numbers are protein IDs corresponding to the 30 DsCEs, with cell death inducing DsCEs in bold font. For reference, the positions of the dothistromin biosynthesis genes [1] are also shown (chromosome 12, grey labels). Within the inner rings, grey bars represent the 875 genes encoding putatively secreted proteins, and green bars (innermost) represent the 397 *in planta*-expressed (>50 Reads Per Million per Kilobase) secreted proteins. The figure was created using CIRCOS (http://circos.ca/software/[3]).

References for Figure S1:

1 de Wit, P. J. G. M. *et al.* The genomes of the fungal plant pathogens *Cladosporium fulvum* and *Dothistroma septosporum* reveal adaptation to different hosts and lifestyles but also signatures of common ancestry. *PLoS Genetics* **8**, e1003088 (2012).

2 Ohm, R. A. *et al.* Diverse lifestyles and strategies of plant pathogenesis encoded in the genomes of eighteen Dothideomycetes fungi. *PloS Pathogens* **8**, e1003037-e1003037 (2012).

3 Krzywinski, M. *et al.* Circos: an information aesthetic for comparative genomics. *Genome Research* **19**, 1639-1645 (2009).

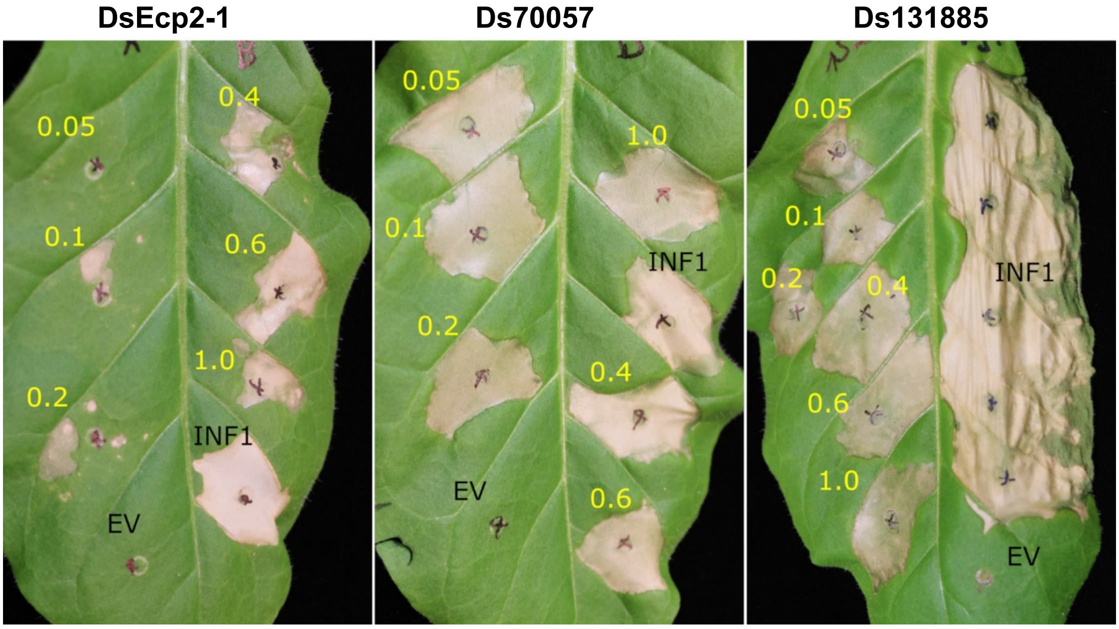

Figure S2: Optical density range trial of *Agrobacterium tumefaciens* cultures. Concentration thresholds are indicated for cell death triggering by DsEcp2-1, but not Ds70057 and Ds131885. The yellow numbers show the used culture OD_{600}; the positive (INF1) and negative (EV) controls were infiltrated at an OD_{600} of 0.6. In the Ds131885 panel, INF1 was trialled at 0.05 to 0.6 (top to bottom).

(a)

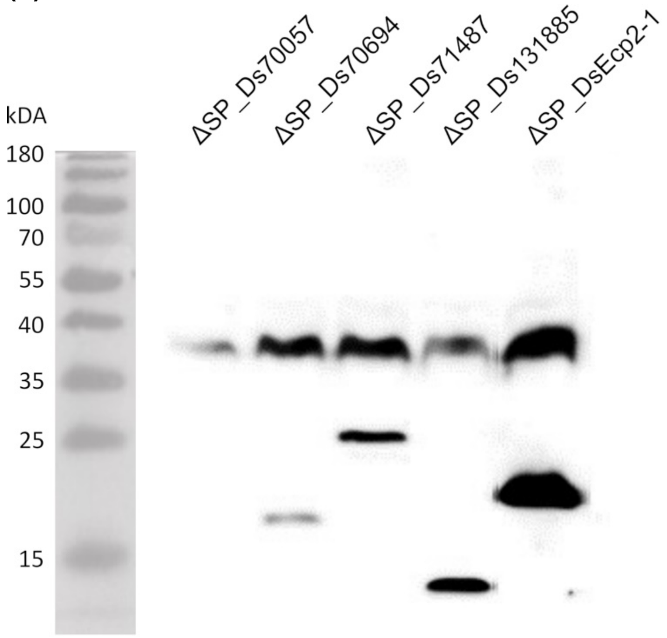

(b)

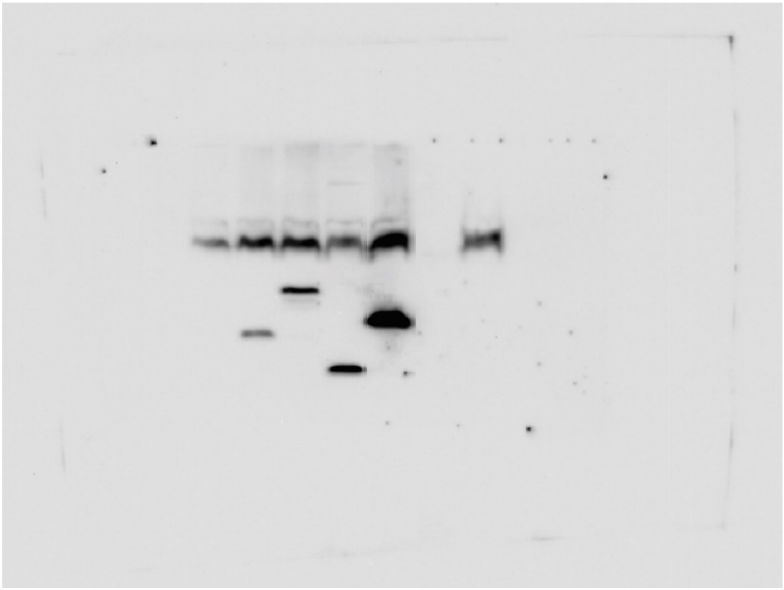

Figure S3: **Western blots of *Dothistroma septosporum* candidate effector proteins.**

(a) Western blots showed that DsCEs triggering cell death in *Nicotiana benthamiana* were expressed in the plant tissue regardless of the presence of a secretion signal peptide and absence of a cell death response (constructs with deleted signal peptide sequences are shown). Immuno-detection was based on primary anti-FLAG antibody. The size marker on the membrane (shown on the left) was a PageRuler™ Prestained Protein Ladder, 10 to 180 kDa (ThermoScientific) and photographed separately from the immuno-detection. (b) Immuno-detection pattern on the full-sized membrane

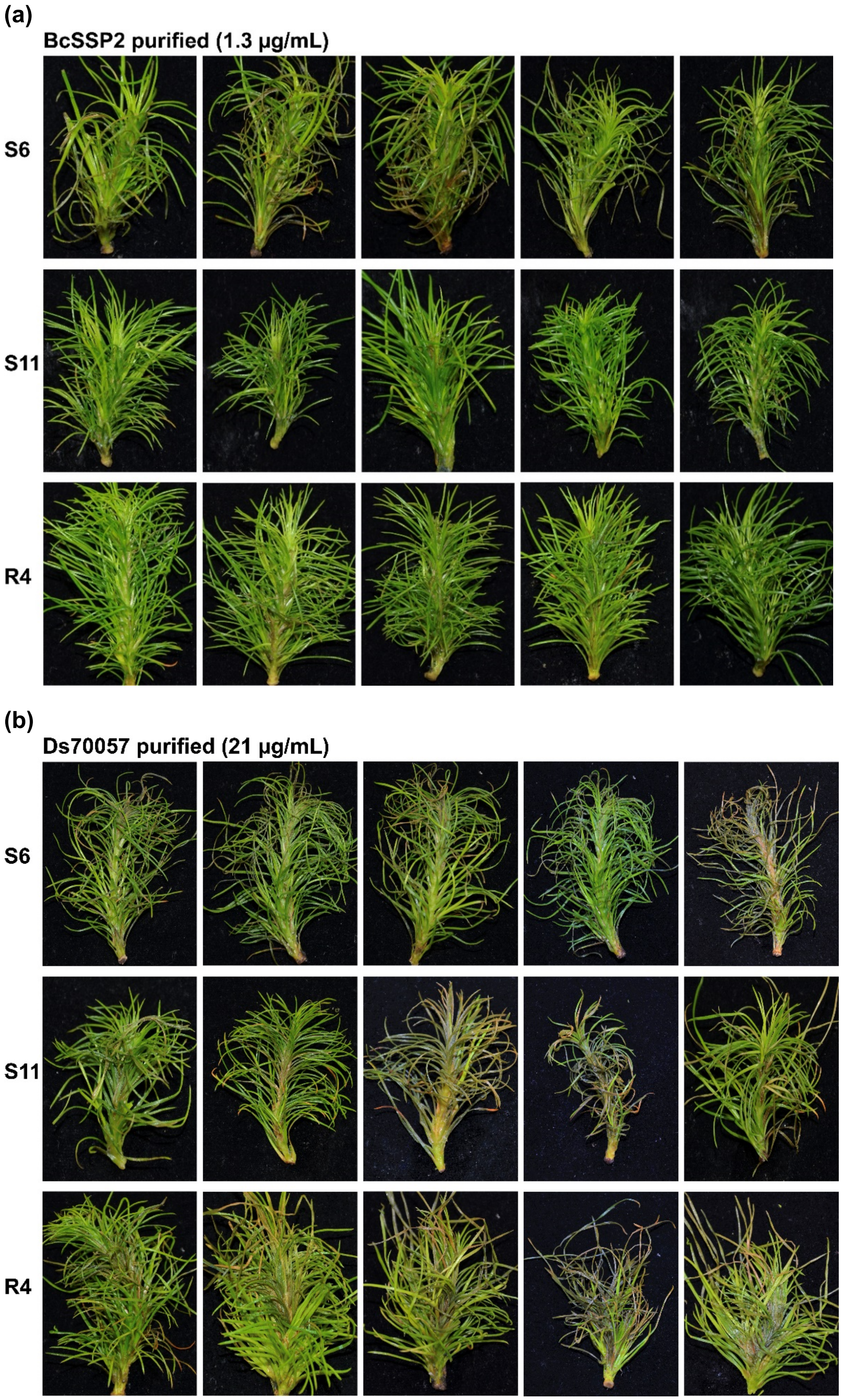

Figure S4: **Replicates of *Pinus radiata* shoot tissue infiltrated with candidate effector proteins.** BcSSP2 (a) and Ds70057 (b) were produced by heterologous expression in *Pichia pastoris*. Photos were taken 7 days after infiltration.
